# Ultrafast demagnetization in bulk nickel induced by X-ray photons tuned to Ni *M*_3_ and *L*_3_ absorption edges

**DOI:** 10.1038/s41598-023-50467-9

**Published:** 2024-01-04

**Authors:** Konrad J. Kapcia, Victor Tkachenko, Flavio Capotondi, Alexander Lichtenstein, Serguei Molodtsov, Przemysław Piekarz, Beata Ziaja

**Affiliations:** 1 Institute of Spintronics and Quantum Information, Faculty of Physics, Adam Mickiewicz University in Poznań, Uniwersytetu Poznańskiego 2, 61614 Poznań, Poland; 2 Center for Free-Electron Laser Science CFEL, Deutsches Elektronen-Synchrotron DESY, Notkestr. 85, 22607 Hamburg, Germany; 3 European XFEL GmbH, Holzkoppel 4, 22869 Schenefeld, Germany; 4 Elettra-Sincrotrone Trieste S.C.p.A, 34149 Trieste, Basovizza Italy; 5 Institute of Theoretical Physics, University of Hamburg, Notkestr. 9-11, 22607 Hamburg, Germany; 6 Institute of Experimental Physics, TU Bergakademie Freiberg, Leipziger Strasse 23, 09599 Freiberg, Germany; 7 Center for Efficient High Temperature Processes and Materials Conversion (ZeHS), TU Bergakademie Freiberg, Winklerstrasse 5, 09599 Freiberg, Germany; 8 Institute of Nuclear Physics, Polish Academy of Sciences, W.E. Radzikowskiego 152, 31-342 Kraków, Poland

**Keywords:** X-rays, Ferromagnetism, Magnetic properties and materials

## Abstract

Studies of light-induced demagnetization started with the experiment performed by Beaupaire et al. on Ni. Here, we present theoretical predictions for X-ray induced demagnetization of nickel, with X-ray photon energies tuned to its $M_3$ and $L_3$ absorption edges. We show that the specific feature in the density of states in the *d*-band of Ni, i.e., a sharp peak located just above the Fermi level, strongly influences the change of the predicted magnetic signal, making it stronger than in the previously studied case of X-ray demagnetized cobalt. It impacts also the value of Curie temperature for Ni. We believe that this finding will inspire dedicated experiments investigating magnetic processes in X-ray irradiated nickel and cobalt.

## Introduction

Ultrafast control of magnetization with lasers remains a hot topic in laser and solid-state physics communities. Apart from traditional terahertz and optical lasers, the state-of-the-art extreme ultraviolet/soft X-ray (XUV) or X-ray free-electron lasers^1–5^ are now also used for demagnetization studies. The main advantage of these lasers is the possibility to resonantly excite core electrons to the magnetically sensitive *d*-band. This X-ray induced electronic excitation changes the population of spin-up and spin-down electrons in the band. As the electronic occupation in the *d*-band determines the magnetization of the material, this results in the decrease of the total magnetic moment in the material^6–8^. In our previous studies^6,7^, we modeled the experimentally observed ultrafast decrease of magnetic small-angle X-ray scattering (mSAXS) signal from X-ray irradiated cobalt which reflected a transient decrease of the cobalt magnetic moment. The XSPIN simulation tool was developed to follow the progressing demagnetization. Our studies have shown that the signal decrease can be explained by ultrafast electron-driven demagnetization.

In this paper, we will apply our model to another widely-used magnetic material, nickel. Magnetic moments of nickel and cobalt are $0.66\, \mu _B$ and $1.70\, \mu _B$ respectively^9^. As nickel’s Curie temperature (627 K)^10^ strongly differs from that of cobalt (1400 K), such study can reveal a potential effect of the Curie temperature on the demagnetization dynamics. Visible spectrum or near-infrared (VIS/NIR) laser-triggered demagnetization of nickel has been studied in various papers, see, e.g.,^11–17^. Interestingly, so far, we have not found relevant experimental data on X-ray pumped Ni demagnetization recorded at X-ray free-electron laser (XFEL) facilities. Therefore, the actual comparison between Co and Ni demagnetization will be performed with theoretical predictions only.

### Simulation scheme

As in our previous works^6–8^, we will use our recently developed XSPIN code to obtain predictions for the “magnetic signal” from the X-ray irradiated nickel. The electronic density of states is obtained from the density functional theory (DFT) calculations implemented in the Vienna Ab initio Simulation Package (VASP)^18–20^. Average absorbed doses considered in the simulations are chosen not to cause structural changes (atomic dislocations) in the irradiated materials. Therefore, the equilibrium density of states (DOS) can be used throughout the whole simulation (the ”frozen atom” assumption). The occupations of electronic levels change during the material exposure to the X-ray pulse, as due to the photoionization, impact ionization and Auger processes, excited electrons leave the band to the continuum. Later, they relax back to the band. As the electrons are heated up by the pulse, they remain hot on femtosecond timescales considered in this study, because their temperature can only decrease through an exchange with the lattice which follows on longer ((sub)ps) timescales. Moreover, due to the assumed common thermalization of all electrons, both spin-up and spin-down electrons follow a Fermi Dirac distribution with a common temperature and a chemical potential. Therefore, the numbers of spin-up and spin-down electrons differ from the corresponding values in the initial state. This thermalization-induced spin flip process, changing the population of spin-up and spin-down electrons, leads to a change of the magnetic signal.

For the simulation, we use a simulation box with $N = 512$ Ni atoms. We performed averaging over $100\ 000$ realizations in the Monte Carlo module of the XSPIN code^6–8^. The XFEL pulse is assumed to have a Gaussian temporal profile of the duration of 70 fs FWHM (full width at half maximum) for *M*-edge case ($M_3$, $E_{\text {edge}} = 66.2$ eV) and 50 fs FWHM for *L*-edge case ($L_3$, $E_{\text {edge}} = 852.7$ eV). The specific pulse duration was chosen to compare the XSPIN predictions for nickel with our previous results for cobalt presented in^6–8^. For more details on the simulation parameters, see Table 1.

**Table 1:** Simulation parameters used in the present work (for nickel).

Region	Edge energy $E_{\text {edge}}$	Photon energy $\hbar \omega _\gamma$	Probed level $\hbar \omega _0$	$\Delta$	Energy range $[\hbar \omega _0 - \Delta ; \hbar \omega _0 + \Delta ]$
*M*-edge	66.2	67	0.8	0.7	[0.1; 1.5]
*M*-edge	66.2	68	1.8	0.7	[1.1; 2.5]
*L*-edge	852.7	853	0.3	1.0	$[-0.7; 1.3]$
*L*-edge	852.7	854	1.3	1.0	[0.3; 2.3]

The position of the probed level, $\hbar \omega _0$, is determined by the energy of incoming photons, $\hbar \omega _{\gamma }$, and the position of the absorption edge $E_{\text {edge}}$, cf. also Eq. (1). Parameter $\Delta$ corresponds to the respective half-width of the *p* band (3*p* band for the *M*-edge case or 2*p* band for the *L*-edge case) and determines the number of states probed in the 3*d* band. All energies are in electronvolts (eV).

**Figure 1:**
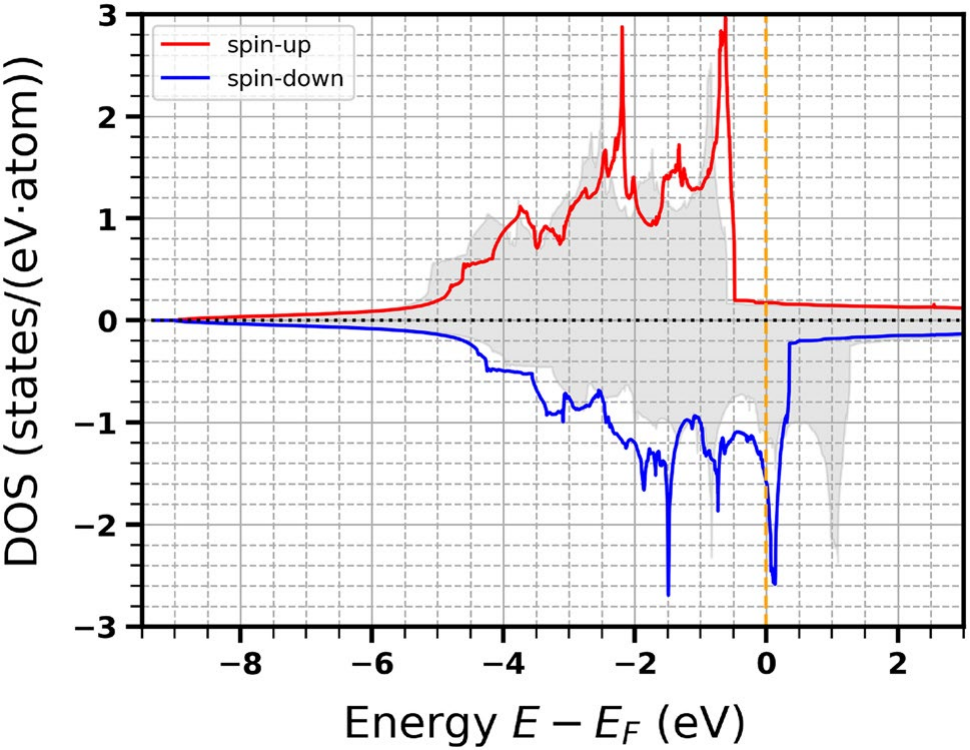
Calculated density of states (in: states per eV, per atom) for fcc nickel. The red and blue lines correspond to DOS for spin-up and spin-down electrons. The grey shaded area indicates the density of states for fcc cobalt investigated in Refs.^6–8^.

## Results

### Spin-polarized electronic density of states from density functional theory calculations

In order to obtain spin-polarized electronic density of states for bulk nickel, we performed first-principle calculation, using the projector augmented-wave (PAW) potentials^21^ and the generalized gradient approximation (GGA) in the Pardew, Burke, and Ernzerhof (PBE) parametrization^22^, implemented in the VASP code^18–20^.

**Figure 2:**
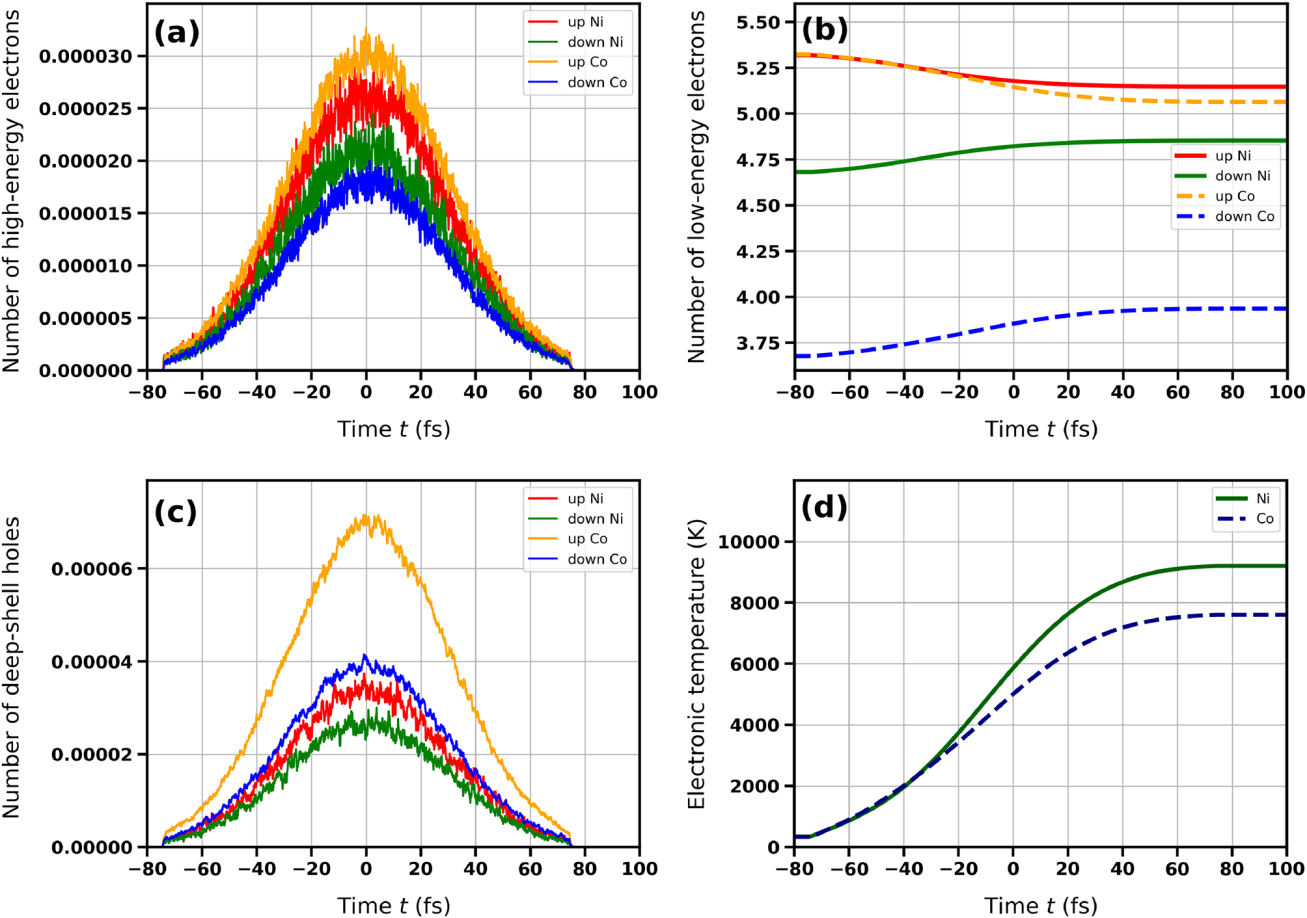
Transient distributions of excited electrons and holes obtained with XSPIN code for X-ray irradiated nickel and for cobalt (as labelled). (**a**) The transient number of polarized high energy electrons (with energies $> 15$ eV), (**b**) the number of low energy electrons (with energies $< 15$ eV), (**c**) the transient number of deep shell holes (with indicated polarization of electrons previously occupying the holes), and (**d**) electronic temperature. The X-ray photon energy was tuned to *M*-edge of Ni ($\hbar \omega _{\gamma } = 68$ eV) and to *M*-edge of Co ($\hbar \omega _{\gamma } = 61.1$ eV). Pulse duration was 70 fs FWHM for both cases. Average absorbed dose was 0.93 eV/atom. Temporal Gaussian X-ray pulse was centered at $t = 0$ fs.

For the summation over the reciprocal space, we used $27 \times 27 \times 27$ Monkhorst-Pack *k*–point grid^23^. The spin-polarized density of states for fcc bulk Ni (calculated for the experimental bulk value of the lattice constant, $a = 3.524\, {\text{\AA }}$) is presented in Fig. 1. It is in an agreement with other DFT calculations (see also, e.g.,^24^). For comparison, the density of states for fcc bulk Co (with $a = 3.545\, {\text{\AA }}$^25^) used in Refs.^6,7^ is also presented. The calculated magnetic moments of nickel and cobalt are $0.62\, \mu _B$ and $1.61\, \mu _B$, respectively, i.e., with a good agreement with those from^9^. Knowing the total spin-dependent density of states $D_{\sigma }(\varepsilon )$ for the system investigated (i.e., not normalized per number of atoms), one can determine the energy, $E_{i,\sigma }$, of the *i*-th level for $\sigma$ electrons from the formula, $i = \int _{-\infty }^{E_{i,\sigma }}d \varepsilon D_{\sigma}(\varepsilon )$. These levels are later used as band levels (details in Refs.^6,7^).

### Electronic properties of X-ray irradiated nickel

Below we present the results on the transient distributions of excited electrons and holes obtained with the XSPIN code for nickel and for cobalt (cf. also^6,7^) irradiated with X rays tuned to their M absorption edges ($\hbar \omega _{\gamma } \sim 68$ eV and $\hbar \omega _{\gamma } \sim 61$ eV respectively; comparably distant from the absorption edge). Figure 2 shows: (a) the transient number (per atom) of polarized high-energy electrons (with energies $> 15$ eV), (b) the number (per atom) of low-energy electrons (with energies $< 15$ eV), (c) the transient number (per atom) of deep shell holes (with indicated polarization of electrons previously occupying the holes), and (d) electronic temperature.

**Figure 3:**
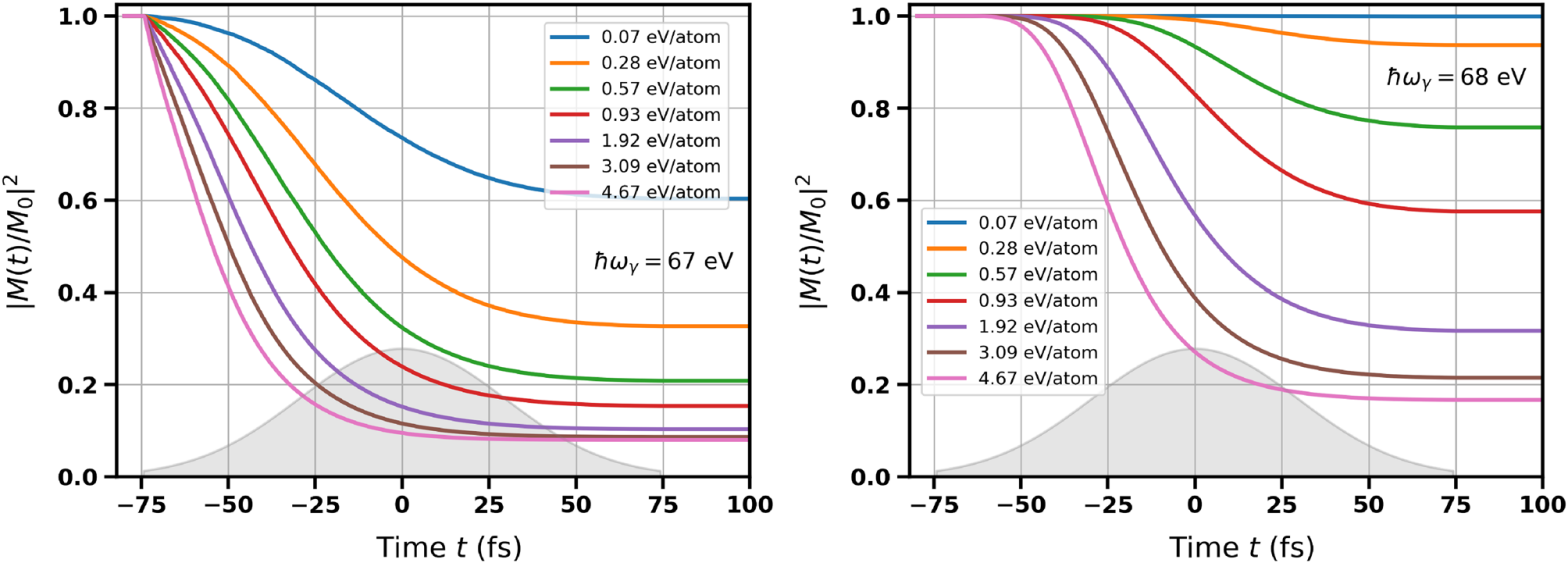
Time dependence of squared normalized magnetization in bulk nickel obtained for the incoming photon energies close to the $M_3$-edge of Ni: $\hbar \omega _{\gamma } = 67$ eV (left) and $\hbar \omega _{\gamma } = 68$ eV (right). The curves obtained for different average absorbed doses are shown (as labelled). The parameter $\Delta$ was equal to 0.7 eV. The grey contour schematically depicts Gaussian temporal pulse profile, which was centered at $t = 0$ fs.

**Figure 4:**
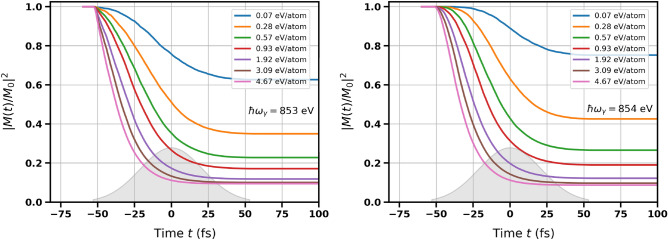
Time dependence of squared normalized magnetization in bulk nickel obtained for the incoming photon energy close to the $L_3$-edge of Ni: $\hbar \omega _{\gamma } = 853$ eV (left) and $\hbar \omega _{\gamma } = 854$ eV (right). The curves obtained for different average absorbed doses are shown (as labelled). The parameter $\Delta$ was equal to 1.0 eV. The grey contour schematically depicts Gaussian temporal pulse profile, which was centered at $t = 0$ fs.

The cutoff energy of 15 eV separates two populations of electrons: (i) low-energy electrons which are band electrons described by the Fermi distribution and (ii) high-energy electrons which are treated as free electrons, with their kinetics described using Monte Carlo scheme. In fact, the cutoff parameter should be large enough to provide a sufficient number of energy levels in the band needed to properly describe high-temperature tail of the Fermi distribution of electrons in the low-energy domain. On the other hand, it cannot be too large, in order to keep the computational costs possibly low. For earlier simulations performed with the XSPIN and XTANT codes, dedicated convergence tests showed that a reasonable choice of cutoff energy is 10–15 eV. The increase of this value does not affect obtained results, which was also confirmed in previous publications^26,27^.

The photoexcitation dynamics in Co and Ni look qualitatively similar, with a stronger excitation in Co (Fig. 2a,c) than in Ni. Collisional relaxation in Ni is also weaker than in Co (Fig. 2b), which leads to the higher electronic temperature in Ni, when compared to Co (Fig. 2d).

### Generalized transient magnetization

In order to follow changing magnetic properties of irradiated materials, we have introduced in Ref.^6^ a generalized transient magnetization which reflects the disparity between electronic populations in spin-up and spin-down electronic subsystems in the *d*-band (cf. also Refs.^28,29^):

1 $$M(t) = \sum\limits_{{\hbar \omega _{0} - \Delta }}^{{\hbar \omega _{0} + \Delta }} {\left[ {N_{ \uparrow }^{{\text{h}}} (E_{{i, \uparrow }} ) - N_{ \downarrow }^{{\text{h}}} (E_{{i, \downarrow }} )} \right]},$$

where $N^{\text {h}}_{\sigma } (E_{i,\sigma })$ denotes the number of empty states at the $E_{i,\sigma }$ level. The probed region in *d*-band extends between $\hbar \omega _0 - \Delta$ and $\hbar \omega _0 + \Delta$, where $\hbar \omega _0 = \hbar \omega _\gamma - E_{\text {edge}}$. Here, $\hbar \omega _\gamma$ is the incoming photon energy, and $E_{\text {edge}}$ is the energy of the resonant core *p*-level. The summation goes here over discrete levels. The parameter $\Delta$ is the *p*-level half-bandwidth, which determines the number of states probed in the 3*d* band, see Table 1. Note that we neglect the subleading effect of the different coupling of polarized light to spin-up and spin-down electrons (XMCD) here. Electronic populations are calculated, assuming the Fermi-Dirac distribution of electrons. Knowing at every time step *t* electronic temperature $T_{\text {e}}$ and electronic chemical potential $\mu$, we have $N^{\text {h}}_{\sigma } (E) = 1 - N_{\text {e},\sigma }^{\text {low}}(E)$ and $N_{\text {e},\sigma }^{\text {low}}(E) = 1/\left\{ 1 + \exp \left[ (E-\mu )/k_{\text {B}} T_{\text {e}} \right] \right\}$. The magnetization defined in Eq. (1) is used, similarly as in^6^, to construct a theoretical equivalent of the experimental observable called magnetic small-angle X-ray scattering (mSAXS) signal strength (Eq. (2)). The resonant magnetic scattering experiment can namely probe electronic occupations of the magnetically sensitive 3*d* band only within an energy region determined by the position and width of the core resonant absorption level (here 2*p* or 3*p*) and the incoming X-ray photon energy. The probing is done by exciting electrons from the resonant absorption level to the 3*d* band. The number of available holes within the probed energy interval of the 3*d* band then determines the strength of the recorded magnetic signal. Namely, the more holes in the interval the more electrons can be excited to it. These electrons then deexcite, emitting mSAXS photons. This implies that if there are no free holes in the probed 3*d* region, the mSAXS signal will not be emitted. Such a definition is a generalization of the standard definition of magnetization, where, for convenience, we calculate the difference between the unoccupied states (holes), instead of the difference between the occupied states (electrons).

Time evolution of the squared generalized magnetization $M^2(t)$ (normalized to its initial value $M_0 = \lim _{t\rightarrow -\infty } M(t)$ before the exposure start; cf. also (1)) for different absorbed doses is presented in Figs. 3 and 4. The values of $\Delta$ in Ni were taken from experimental measurements. They are: $\Delta = 0.7$ eV for nickel *M*-edge^30–32^ and $\Delta =1.0$ eV for nickel *L*-edge^33–36^. One can see that the decrease of magnetization becomes stronger with the increasing absorbed dose, and also strongly changes with the incoming photon energy in the vicinity of the absorption edge. Interestingly, if the probed region in *d*-band includes the sharp peak in the DOS of spin-down electrons near the Fermi level (X-ray photon energies of 67 eV and 853 eV for *M*- and *L*-edge respectively; see Table 1), the observed magnetization change is much stronger than in case when this peak is not included (X-ray photon energies of 68 eV and 854 eV for *M*- and *L*-edge respectively). The reason is that the peak ”provides” a large number of unoccupied states for the resonant excitation from *p*-level, which leads to a stronger decrease of the transient magnetization.

Note that the decrease of magnetization is stronger for Ni than for Co (cf. Fig. 4 from Ref.^6^ and Fig. 3 from Ref.^7^ at the absorbed dose of 0.93 eV/atom). The reason is that cobalt DOS does not show such a pronounced peak close to the Fermi level, and the reduction of magnetization is, therefore, suppressed. This can also explain the lower Curie temperature for nickel than for cobalt.

### Calculation of the mSAXS signal

**Figure 5:**
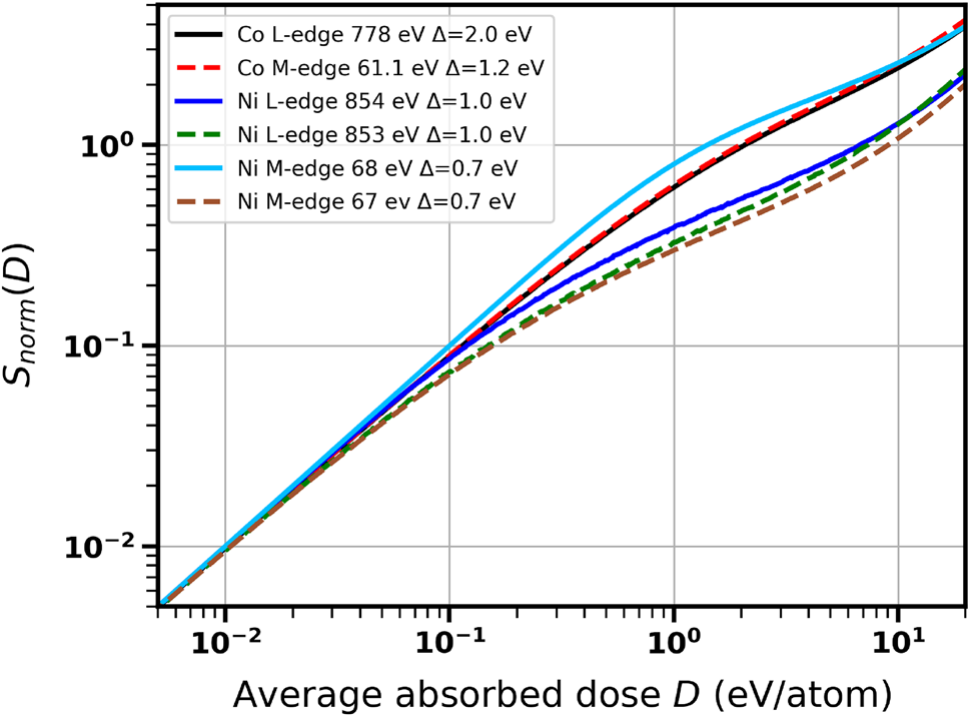
Scattering efficiency $S_{\text {norm}}$ for nickel and for cobalt as a function of average absorbed dose for X-ray photon energies close to the *M* and *L* absorption edges of Ni and Co. Results are presented for various incoming photons energies $\hbar \omega _{\gamma }$ and parameters $\Delta$ (as labelled).

Similarly as in Ref.^6^, we can calculate the magnetic small-angle X-ray scattering signal strength from the generalized magnetization. If such an mSAXS experiment would be conducted, the polarization of light should be circular in order to obtain the strongest mSAXS signal. Similarly as in the mSAXS experiment by A. Philippi-Kobs et al.^37^ for cobalt, which results were analyzed in^6^, the X rays would then arrive at a normal incidence to the Ni layer. The magnetization of Ni should be out-of plane, i.e., perpendicular to the layer. The signal strength then is obtained as:

2 $$\begin{aligned} S = a \int M^2(t) I(t) dt, \end{aligned}$$

where *I*(*t*) is the X-ray pulse intensity and *a* is a proportionality coefficient. Pulse fluence is then: $F = \int I(t) dt$. It is proportional to the average absorbed dose, $D \propto F$, where the proportionality coefficient depends on the material parameters as well as on the photon energy. The dose dependence of the normalized signal strength, $S_{\text {norm}} = S(D)[D_0/S(D_0)]$ for the corresponding experimental $\Delta$ values is presented in Fig. 5. The normalization follows Ref.^6^, with the reference dose, $D_0=10^{-4}$ eV/atom for all considered cases.

Similarly as observed for generalized magnetization, the signal strength strongly depends on the fact if the probed region in *d*-band includes or does not include the sharp peak in the DOS of the spin-down electron fraction near the Fermi level - being distinctly higher in the latter case. This explains also a stronger decrease of $S_{\text {norm}}$ for nickel than for cobalt.

## Conclusions

We provided theory predictions for transient electronic properties in X-ray irradiated Ni at photon energies close to $M_3$ or $L_3$ absorption edges, as well as for the resulting magnetization change and the mSAXS scattering strength. The results obtained indicate the same ultrafast demagnetization mechanism (caused by electronic excitation and relaxation) as in X-ray irradiated cobalt, occurring at a similar timescale. However, due to the difference in the DOS structure of the *d*-band, the degree of demagnetization for an equivalent dose is higher in Ni than in Co. This finding is also consistent with the lower Curie temperature for nickel than for cobalt. We expect that these theory predictions will inspire dedicated experimental studies on ultrafast X-ray induced demagnetization of nickel, a benchmark magnetic material of various applications.

As in our previous studies on Co, we did not consider here atomic motion, and kept electronic band structure unchanged. This assumption does not hold for the case of high absorbed doses which may induce ultrafast structural changes in irradiated materials. A rigorous estimation of the structural damage threshold is difficult at the 100 fs timescale. The ”standard” definition for structural damage threshold in a metal provides a threshold dose for its thermal melting. This dose for nickel is estimated as  0.57 eV/atom. However, the thermal melting would require picosecond times to be completed, as such timescales are needed to transfer sufficient amount of energy from the electronic system to the lattice. The timescale of atomic displacements during the structural transformation would then be longer than the femtosecond pulse duration (see, e.g., Refs.^38–42^). Therefore, at 100 fs timescale, we cannot consider the standard melting dose as an indicator for on-going atomic displacements, and can still assume with a reasonable accuracy that atoms do not change their positions during the simulations, even for doses a few times higher than 0.57 eV/atom^6^.

However, at much higher absorbed X-ray doses or if the model is applied at picosecond timescales (e.g., in order to follow the recovery of the magnetization), the possible atomic relocations should be taken into account. The model should then be developed further, enabling inclusion of atomic dynamics and transient band structure.

## Data Availability

The data presented in this study are available from the corresponding authors (K.J.K.—konrad.kapcia@amu.edu.pl, V.T.—victor.tkachenko@xfel.eu, or B.Z.—ziaja@mail.desy.de) upon reasonable request.

## Code availability

The XSPIN code that supports the conclusions within this paper and other findings of this study is available under a license agreement. The licensor is Deutsches Elektronen-Synchrotron DESY, Notkestr. 85, 22607 Hamburg, Germany. Please contact the corresponding authors for more details.
